# Effect of photobiomodulation in secondary intention gingival wound healing—a systematic review and meta-analysis

**DOI:** 10.1186/s12903-021-01611-2

**Published:** 2021-05-13

**Authors:** Pooya Ebrahimi, Mahdi Hadilou, Ferdos Naserneysari, Amirmohammad Dolatabadi, Rana Tarzemany, Nafiseh Vahed, Leila Nikniaz, Reza Fekrazad, Leila Gholami

**Affiliations:** 1 Student Research Committee, Faculty of Dentistry, Tabriz University of Medical Sciences, Tabriz, Iran; 2 Department of Oral Biological and Medical Sciences, Faculty of Dentistry, University of British Columbia, Vancouver, Canada; 3 Research Center for Evidence-Based Medicine, A Joanna Briggs Institute Affiliated Center, Tabriz University of Medical Sciences, Tabriz, Iran; 4 Emergency Medicine Research Team, Tabriz University of Medical Sciences, Tabriz, Iran; 5 Tabriz Health Services Management Research Center, Faculty of Management and Medical Informatics, Tabriz University of Medical Sciences, Tabriz, Iran; 6 Department of Periodontology, Dental Faculty, Laser Research Center in Medical Sciences, AJA University of Medical Sciences, Tehran, Iran; 7 Department of Periodontics, Dental Research Center, School of Dentistry, Hamadan University of Medical Sciences, Shahid Fahmideh Blvd, 654178-38741 Hamadan, Iran

**Keywords:** Photobiomodulation (PBM), Low-level laser therapy (LLLT), Secondary intention wound healing, Periodontal surgery

## Abstract

**Background:**

Photobiomodulation is widely being used to improve the wound healing process in dentistry and a vast majority of studies have proven its benefits. But there are plenty of knowledge gaps according to the optimal laser characteristics which should be used to maximize the healing effects of lasers. The goal of this systematic review and meta-analysis was to determine the effect of photobiomodulation (PBM) as an adjunctive treatment to periodontal therapies to evaluate secondary intention gingival wound healing and post-operative pain.

**Methods:**

Five databases (PubMed, Embase, Scopus, ProQuest, and Web of Sciences) were searched up to November 30, 2020, for clinical trials that reported the result of the application of PBM on secondary gingival healing wounds and post-operative pain and discomfort after periodontal surgeries. Two independent reviewers selected the eligible studies and the outcomes of interest were extracted. The quality of eligible studies was assessed using the Cochrane Handbook for Systematic Reviews of Interventions.

**Results:**

Ultimately, twelve studies were included in this review. The application of PBM as an adjunct to periodontal surgeries resulted in a significant improvement in wound healing indices. The Landry wound healing index at the 7th post-operative day was significantly improved (SMD = 1.044 [95% CI 0.62–1.46]; p < 0.01) in PBM + surgery groups compared to the control groups. There was also a statistically significant increase in the complete wound epithelialization (RR = 3.23 [95% CI 1.66–6.31]; p < 0.01) at the 14th post-operative day compared to the control groups. The methods used to assess the post-operative pain were heterogeneous, and therefore the results were limited which made the meta-analysis for post-operative pain assessment not possible.

**Conclusion:**

Based on the results of this review, PBM can be effectively used as a method to improve secondary intention wound healing. High-quality randomized clinical trials, however, are needed in the future to identify the optimal PBM irradiation parameters and the effect of PBM on post-operative pain.

**Supplementary Information:**

The online version contains supplementary material available at 10.1186/s12903-021-01611-2.

## Background

Improvement of wound healing after periodontal surgeries is a critical factor in achieving favorable clinical results [1]. Optimal wound healing and reduction in the severity and duration of post-operative pain result in a better prognosis and outcome of the periodontal treatment and patient satisfaction [2]. Post-operative discomfort or pain is a subjective experience, and the process of wound healing is multifactorial [3, 4]. This pain is sometimes associated with a delayed wound healing. It is influenced by several emotional, clinical, and iatrogenic causes including stress and psychological condition, patient's earlier experiences, type and duration of surgery, surgeon’s experience and skills, and also the type of wound closure (primary or secondary) [5–8]. Some of the common medications and methods used by clinicians to improve wound healing after periodontal surgeries include the application of chlorhexidine with or without alcohol [9, 10], nutritional supplementations [11], and antibiotics such as azithromycin [12], vitamin D [13], professional tooth cleaning [14], and the use of fibrin sealants instead of sutures [15].

Secondary intention healing wounds can be associated with considerable discomfort and delayed healing compared to primary intention healing wounds after the periodontal flap surgeries. Gingivectomies, depigmentation procedures, and harvesting free gingival graft tissues from the palatal area are common secondary intention healing wounds. This healing type occurs when the wound site is left open to heal mostly by granulation, contraction, and epithelialization. Moreover, we encounter more scar formations and contraction [8].

The application of photobiomodulation (PBM) as an adjunctive therapy to improve wound healing has attracted the attention of many researchers in recent years [16, 17]. PBM, includes the application of laser or light-emitting diode (LED) beams for stimulation of healing, relieving pain, and reducing inflammation [18]. Numerous studies have shown the positive inductive effects of photobiomodulation on the viability and proliferation of skin and gingival fibroblast cells, in vitro [16, 17, 19, 20]. Therefore, this biophysical approach has been considered as a treatment modality which can stimulate the endogenous healing process. The main mechanisms considered for the observed biological response is the absorbance of low-level light irradiation by cellular photoreceptors or ROS production and subsequent generation of highly reactive, transient biochemical intermediates, changes in cellular ionic gradients or cell polarity and ultimate increase in ATP production, recruitment of transcription factors and increase in cell activity. This results a secondary phase of responses including cell proliferation, differentiation and migration, angiogenesis, production of growth factors and matrix synthesis which contribute to promotion of wound healing [21–24].

Clinically, PBM has also been reported to result in a decreased pain sensation, enhancement of keratinization [25–27], and improvements in periodontal clinical characteristics such as enhancement in clinical attachment level (CAL) and probing depth (PD) [28, 29]. Several studies have emphasized the significant effect of PBM on post-operative pain reduction and wound healing improvement after periodontal surgeries, although there exist some controversies in the reported results [20, 30–34].

The characteristics of PBM irradiation parameters need to be considered as an important factor in order to achieve an optimal dose of irradiation, as a small amount or too high irradiation dose could have no effect or undesirable inhibitory results on wound healing outcomes [35]. Various laser wave lengths and settings have been used to promote oral wound healing and there is a large amount of information about PBM application in the field of wound healing. However, the effects on open oral soft tissue wounds and the most fitting laser characteristics to improve the healing of these types of wounds have not been specified to date. Therefore, the present review, aimed to determine the effectiveness of the application of PBM as an adjunctive treatment in periodontal surgeries to improve secondary intention wound healing and post-operative pain and find an evidence-based answer to this question:

"Does the application of PBM as an adjunct, improve the secondary intention wound healing after periodontal soft tissue surgeries?"

## Methods

### Protocol registration

All study concepts and details were recorded and published in the international prospective register of systematic reviews (PROSPERO). (Registration ID: CRD42020192403).

### Focused question

The Preferred Reporting Items for Systematic Review and Meta-Analysis (PRISMA) [36] guidelines were respected.

The addressed PICO was: "Can photobiomodulation improve soft tissue secondary wound healing and post-operative pain after periodontal surgeries?".

### Selection criteria

The eligibility criteria for studies to be included in this review were based on the following PICOS:

(*Population*): the participants who had undergone periodontal, soft tissue surgeries resulting in a secondary intention healing wound (depigmentation, gingivectomy, or free gingival soft tissue grafts) and without any systemic conditions.

(*Interventions*): the intervention groups that were treated with adjunctive PBM (Laser or LED) irradiation on the gingival wound site after the surgery.

(*Outcomes*): our outcome measures of interest were wound healing parameters such as Landry Wound Healing Indices (WHI), epithelization, and pain after surgery.

(*Study design*): this review was restricted to controlled trials published in English.

All animal studies, opinion articles, in vitro studies, reviews, unpublished studies, abstracts, and articles in which the patients had systemic disease, the wounds were sutured, and interventions including flap elevation were excluded.

### Search strategy

The authors (NV and MH) performed an extensive search in the online databases of Embase, PubMed, Scopus, ProQuest, and Web of Science in search of relevant studies which had been published before 30 November 2020. The literature search was conducted using the modified type or combination of the following words: “photobiomodulation”, “PBM”, “low level laser therapy”, “low intensity laser therapy”, “LLLT”, “low level light therapy”, “low power laser therapy”, “low power laser irradiation”, “periodontal surgery”, “wound healing”, “gingivectomy”, “pigmentation”, “depigmentation”, “palatal donor site” (Additional file 1: Appendix 1). The reference lists of included articles were also manually searched. Gray literature search of evidence was also conducted.

### Screening methods and data abstraction

Two reviewers (AD and FN) independently screened the studies in three steps. The first step was the removal of duplicates. After assessing the remaining studies based upon the titles and abstracts, then, ruling out the irrelevant, the authors reviewed the full texts of selected articles. Full texts were included, considering the eligibility criteria. If there were opposing opinions among the reviewers, they were referred to a third reviewer (LG), then the final decision was made through a group discussion.

Data were extracted from the full text of selected studies for the following factors: author/year, study type, the number of subjects, type and site of the procedure, study groups, evaluated criteria and study outcome, use of analgesics, and follow-ups.

Considering the importance of irradiation parameters in PBM therapies, in another table, the following data concerning the irradiation parameters applied were extracted:

Laser type, wavelength**,** application mode, output power, total exposure time**,** total energy**,** beam diameter or probe spot size, energy density, distance to the intervention site, method of application, frequency of laser treatment, or the number of irradiation sessions.

### Risk of bias among the studies

Evaluation of the risk of bias among the included articles was performed associated with the following concepts by the reviewers:

Random sequence generation, allocation concealment, blinding of participants and personnel, blinding of outcome assessment, incomplete outcome data, selective reporting, and other bias.

To assess the risk of bias in each study, the Cochrane Handbook for Systematic Reviews of Interventions was used [37]. Both of the assessors discussed and resolved any disagreements.

### Data synthesis

The meta-analysis was done using Comprehensive Meta-Analysis (CMA) software version 2. The relative risk (RR) and mean differences (MD) were used for dichotomous and continuous data, respectively, considering a 95% confidence interval. To demonstrate the achieved results, forest plots were utilized. The statistical heterogeneity was recognized by the application of the chi-square test and I^2^ value [38]. To check publication bias and illustrate it as a funnel plot, Egger’s test was done [39].

## Results

### Study selection

**Fig. 1:**
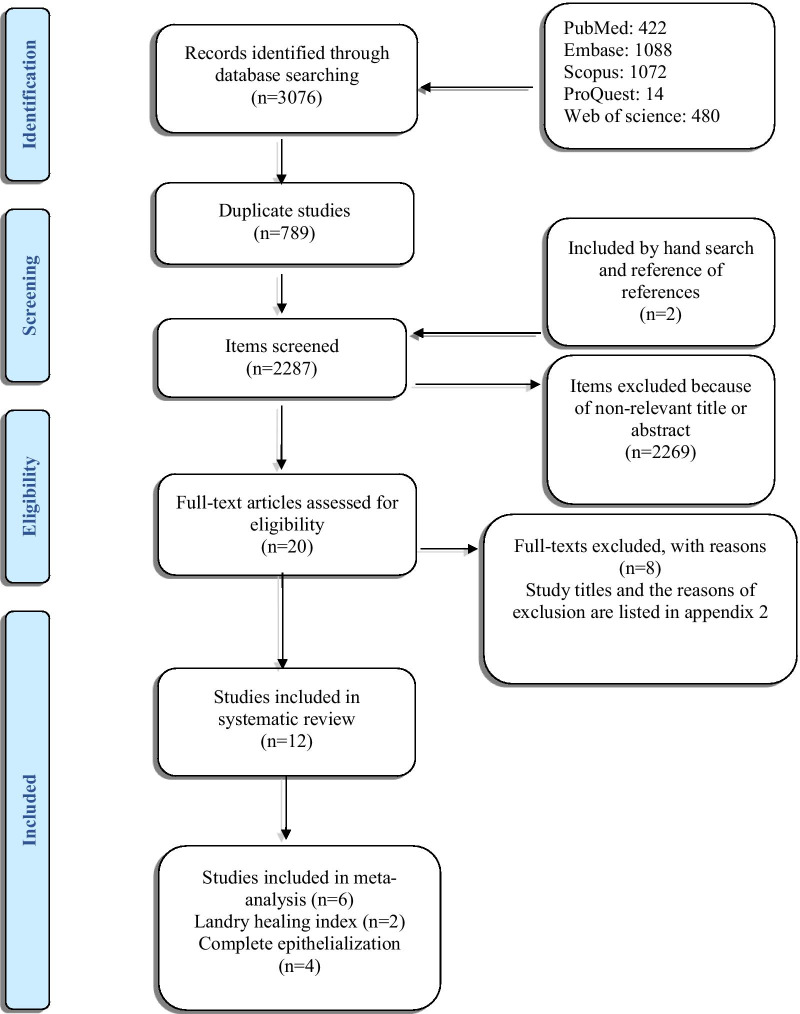
Flow diagram according to preferred reporting items for systematic reviews and meta-analysis (PRISMA)

After the initial search, 3076 studies were found. The authors removed the duplicates (n = 789) and evaluated the titles and the abstracts. A number of 2269 articles were found to be not relevant to the study’s objective and were excluded. Twenty studies were selected for a thorough evaluation of full-texts in which, eight studies were put aside as they did not meet the eligibility criteria (Additional file 2: Appendix 2). Finally, twelve studies were selected as are shown in the study selection flow diagram (Fig. 1) [17, 19, 20, 30, 32–34, 40–44].

### Characteristics of included studies

Table 1 shows the characteristics of the included studies. All of the twelve included studies were clinical trials. Trials were originated from Brazil [30, 32, 33, 40], Iran [19], Turkey [17, 34, 41, 43], and India [20, 42, 44].

**Table 1:** Study characteristics table

References	Study type	Type of procedure/groups	Site of procedure	No. of patients	Follow-up	Evaluated criteria	Use of analgesics	Effect of PBM on Healing	Effect of PBM on pain
Kohale et al. [20]	Split-mouth	Gingival enlargement; gingivectomy G1: Gingivectomy with LLLT G2: Gingivectomy without LLLT	Maxillary and mandibular anterior region (bilaterally symmetrical)	G1:40 G2:40	Days 3, 7, 30	Surface epithelialization: staining with methylene blue Pain: Numerical Rating Scale (NRS) Healing Index scores (HI)	In case of pain; any medication if required	Enhanced wound healing	Reduced post-operative pain and discomfort
Vieira et al. [40]	Parallel	Free gingival grafting; palate donor site G1: Application of LED laser on donor site G2: No additional treatment	NM	G1:5 G2:5	days 1, 2, 3, 4, 5, 6, 7, 14, and 21	Wound healing by peroxide test Pain: VAS	Paracetamol 500 mg (if necessary)	Enhanced wound healing	Reduced post-operative pain and discomfort
Lingamaneni et al. [42]	Split‑mouth	G1: Gingivectomy/Gingivoplasty + Diode laser G2: Gingivectomy/Gingivoplasty	Bulbous or over contoured gingiva in either of the jaws with a minimum of six teeth affected	G1:10 G2:10	Days 3, 7, 14	Healing index by Landry et al Complete epithelialization; staining by a plaque‑disclosing agent (2‑Tone disclosing tablets, Young, USA)	Ibuprofen (in case of pain; but not more than thrice a day.)	Enhanced wound healing	Not Examined
Heidari et al. [19]	Split-mouth	Free gingival grafting; palate donor site G1: Donor site received diode laser G2: Donor site received placebo laser therapy	Palate	G1:12 G2:12	Epithelialization of donor site: days 7, 14, 21, and 30 VAS: in the day of surgery, then every day until day 12	Epithelialization of donor site Clinical Healing (CH), NSAID intake Pain: VAS	Gelofen 400 mg (Jaber EbneHayan, Iran)	Enhanced wound healing	No effect on post-operative pain
Ustaoglu et al. [17]	Parallel	Free gingival grafting; palate donor site G1: PBM of donor site G2: Sham laser application	Palate; canine to the first molar	G1:20 G2:20	Days 3, 7, 14, 21, and 30	Epithelialization: H_2_O_2_ bubbling test Wound Healing Index: WHI Pain: VAS Bleeding, palatal tissue consistency, color match, tissue thickness Number of analgesics	Paracetamol 500 mg (if needed)	Enhanced wound healing	Not Examined
Chawla et al. [44]	Split-mouth	Depigmentation G1: Surgical Stripping + LLLT G2: Surgical Stripping	Sites extending from right canine to the midline and left canine to the midline of the maxilla or mandible	G1:15 G2:15	Days 3, 7, and15	Wound healing: erythrosine solution Pain: VAS	Paracetamol 500 mg (in case of pain; continued maximum for 2 days.)	Enhanced wound healing	No effect on post-operative pain
Keskiner et al. [43]	Parallel	Free gingival grafting; palate donor site G1: PBM on donor site G2: PBM sham on donor site	Palate; canine to the first molar	G1:15 G2:15	days 7, and 12	TGF-b1, PDGF-BB, and IL-8 levels	NM	Enhanced wound healing	Not Examined
Ozcelik et al. [41]	Split-mouth	Gingival hyperplasia; Gingivectomy and gingivoplasty G1: Adjunctive LLLT in one side G2: No additional treatment	Gingiva at the maxillary or mandibular anterior region	G1:20 G2:20	days 1, 3, 7, and 15	Wound healing: disclosing solution (Mira-2-tone, GMBH & Co., Duisburg, Germany) Swelling, bleeding, edema, plaque	Naproxen sodium	Enhanced wound healing	Not Examined
Amorim et al. [30]	Split-mouth	Gingivectomy G1: Adjunctive LLLT in one side G2: No additional treatment on the other side	Bilateral mandibular and maxillary bicuspid teeth	G1:20 G2:20	Immediately post-operative and at days 3, 7, 14, 21, 28, and 35	Wound healing examined by three periodontists Attached gingiva, probing depth, keratinized gingiva	NM	Enhanced wound healing	Not Examined
Isler et al. [34]	Parallel	Free gingival grafting; palate donor site G1: Diode laser PBM G2: Ozone therapy G3: Neither PBM nor ozone therapy	Palate	G1:12 G2:12 G3:12	Days 1, 2, 3, 7, 15, and 30	Wound healing: 3% hydrogen peroxide Pain: VAS	Flurbiprofen 100 mg tablets (Majezik; Sanovel, Istanbul, Turkey) (in case of pain; up to thrice a day for a week)	No significant improvement	Reduced post-operative pain and discomfort
Damante et al. [32]	Split-mouth	Gingivoplasty G1: Diode laser LLLT G2: No irradiation	Keratinized mucosa regions of teeth 11, 12, and 13 or 41, 42, and 43	G1:16 G2:16	days 7, 14, 21, and 60	Incisional biopsies: morphometric analysis of the gingival epithelial and connective tissue	NM	No significant improvement	Not Examined
Damante et al. [33]	Split-mouth	Gingivoplasty G1: Diode laser LLLT G2: No irradiation	Keratinized mucosa regions of teeth 11, 12, and 13 or 41, 42, and 43	G1:11 G2:11	Days 7, 15, 21, 30, and 60	Gingival color, texture, and contour	NM	No significant improvement	Not Examined

*LLLT* low-level laser therapy, *NM* not mentioned, *G* group, *PBM* photobiomodulation

The type of procedure in four of the included studies was gingivectomy [20, 30, 41, 42], it was gingival grafting in five studies [17, 19, 34, 40, 43], gingivoplasty in two studies [32, 33], and one study used surgical stripping for gingival hyperpigmentation [44]. The overall number of participants among the studies ranged between 10 and 40. In all studies, smoking history was absent, and the follow-up frequencies were between 2 and 13 times (Table 1).

### Assessment of risk of bias among the studies

**Fig. 2:**
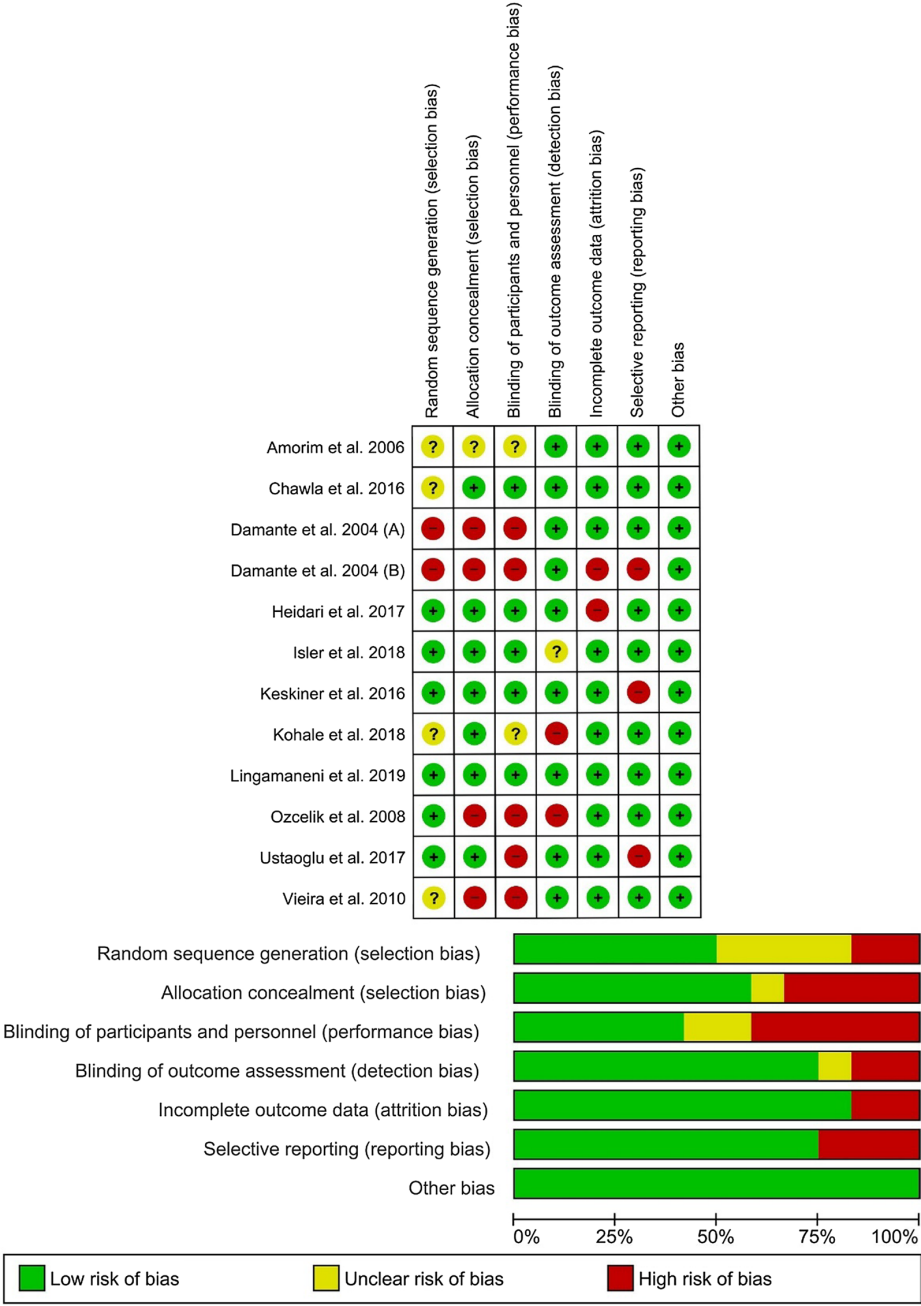
Risk-of-bias analysis: **a** Risk of bias summary; **b** risk of bias graph

The summary of the risk of bias is shown in Fig. 2. Seven articles were found to have issues regarding randomization or concealment of allocation [20, 30, 32, 33, 40, 41, 44] (selection bias). The main cause of bias in the included studies was related to blinding. Five items did not blind the participants or the personnel [17, 32, 33, 40, 41], and it was unclear in two studies [20, 30] (performance bias). Also, two studies did not blind the outcome assessor [20, 41] and it was unclear in one study [34] (detection bias). Also, four studies had a bias in reporting [17, 19, 33, 43] (attrition or reporting bias).

### Irradiation parameters

Table 2 shows the characteristics of the used lasers or LEDs in the included studies. The laser types applied in 11 studies were diode [17, 19, 20, 30, 32–34, 41–44] and one study used LED [40]. The frequency of irradiation sessions was around 3–8, and a 588 to1064nm range of laser wavelengths were used. Power output and total irradiation time range were 15–5000 mW and 32 to 2400 s, respectively. The total energy range applied to the wound area was 12–1200 J; however, some studies had not mentioned this parameter. Energy density ranged from 1.6 to 8.6 J/cm^2^. Six studies had used the laser in a non-contact method with a distance of 1 mm to 2 cm to the surface of the tissue [17, 20, 34, 42–44], and the other five studies used the laser in contact with the tissue [19, 30, 32, 33, 41].

**Table 2:** Laser characteristics table

References	Type of laser	Wavelength (nm)	Mode	Output power (W)	Total exposure time (s) (n × t) Exposure time per point/session	Total energy (J)	Beam diameter (µm-mm)/Probe spot size (cm^2^)	Energy density (J/cm^2^)	Distance to intervention site	Method of application	Frequency of laser treatment/Number of irradiation sessions
Kohale et al. [20]	Diode InGaAsP (Biolase—iLase‑7400040‑0XX)	940	Continuous	0.1	360 (120 × 3) (40 per point × 3 points 120 s in each session)	12	1 cm^2^	4 per teeth (3 points)	1–2 cm	In a circular motion 3 points were irradiated in each session 1cm^2^ area over each tooth	Instantly post-surgery, 3, and 7 days later/3
Vieira et al. [40]	LED (details not mentioned.)	650	NM	5	NM	NM	NM	4 per point (2 points)	NM	A biostimulation probe was used on the surgical site	Instantly post-surgery, 48 h, and 72 h later/3
Lingamaneni et al. [42]	Diode (λ = 810 nm Picasso diode laser, AMD lasers, Indianapolis, USA)	810	Continuous	0.1	1200 (4** × **300) 300	120	NM	NM	Noncontact (not mentioned the value)	Test site was exposed with a diode laser at a power of 0.1 W; used in a continuous wave, non-contact mode	Instantly post-surgery, 7, and 14 days later/4
Heidari et al. [19]	Diode (THOR® Laser, London, UK)	660	Continuous	0.2	160 (5** × **32) 32	32	5 mm	4 per point (8 points)	Slight contact	The laser was directed vertically with minor contact with the intervention site	Instantly post-surgery, 1, 2, 4 and 7 days later/5
Ustaoglu et al. [17]	Diode GaAlAs (Calibrated by Ezlase; Biolase_ Technology, Inc., _Istanbul, Turkey)	940	Continuous, the pulse interval size was 0.10 ms, and the pulse length was 0.05 ms	3	32 (4** × **8) 8	Total applied energy was 24 J (energy over the actual wound area was 7.2 J)	2.8 cm^2^	8.6	1 mm	The handpiece was situated vertically at a distance of 1 mm at the top of the intervention area	4 times (48 h interval)
Chawla et al. [44]	Diode (Details not mentioned)	810	Continuous	1	1200 (4** × **300)	1200	NM	NM	1 mm	To avoid the scattering of the beam to the contrary side, stents were prepared prior to the operation using putty impression material to conceal the control sites during the PBM procedure	LLLT procedure was repeated each day until the 7th day
Keskiner et al. [43]	Nd:YAG (Fotona Fidelis III, Ljubljana, Slovenia)	1064	Continuous	0.25	50 (5** × **10)	12	0.28cm^2^	1.6 per point (5 points)	1 cm	A biostimulation probe was used on the surgical site	Instantly post-surgery, first, second, third, and fourth days later/5
Ozcelik et al. [41]	Diode (ULOCKS, VSMA Lab, Voronezh, Russia)	588	Continuous	0.12	2400 (8** × **300)	288	NM	4	Contact	PBM was used on one side of the surgical area with minor contact	Instantly post-surgery, and daily for 7 days/8
Amorim et al. [30]	Diode (Model IR 500; Laser Beam, Rio de Janeiro, Brazil)	685	Continuous	0.05	320 (4** × **80)	16	2 mm	4	Contact	The irradiation was made while holding the delivery tip perpendicular to the tissue surface	Instantly post-surgery, 24 h, 3rd, and 7th days/4
Isler et al. [34]	Diode ( λ = 970 ± 15 nm, 14 W source power; SIRO Laser Xtend, Sirona Dental Systems GmbH, Bensheim, Germany)	970	Continuous	2	120 (4** × **30) 30	240	4 mm	5.25 per point (5 points)	1 mm	A biostimulation probe was used on the test site	Instantly post-surgery, 1, 3, and 7 days later/4
Damante et al. [32]	Diode GaAlAs	670	NM	0.015	NM	NM	4 mm	4 per point (3 points)	Contact	Diode laser was used in the punctual mode with slight contact to three points	Every 48 h for 1 week, for a total of four sessions/4
Damante et al. [33]	Diode GaAlAs	670	NM	NM	NM	NM	4 mm	4 per point (3 points)	Contact	Diode laser was used in the punctual mode with slight contact to three points	Every 48 h for 1 week, for a total of four sessions/4

*NM* not mentioned, *nm* nanometer, *W* watt, *s* seconds, n number, t times, *J* joules, *µm* micrometer, cm^2^ square centimeter, *mm* millimeter, *LED* light-emitting diode, *LLLT* low-level laser therapy, *PBM* photobiomodulation

### Main outcomes of the studies

#### Wound healing

Several wound healing parameters evaluated in the included studies, included degree of epithelialization, healing index (HI), clinical healing (CH), Landry wound healing index (WHI), color match, tissue thickness (TT) and scar, tissue remaining wound area (RWA), tissue color and contour, and incisional biopsies for histological examinations [17, 19, 20, 30, 32–34, 40–44] (Table 1). Nine studies out of twelve [17, 19, 20, 30, 40–44] reported a significant improvement in wound healing parameters after PBM application. Damante et al. [32, 33] and Isler et al. [34] reported no stimulatory effect of PBM on wound healing. The number of included articles was not adequate to evaluate the publication bias using a funnel plot [46].

Four studies evaluated the degree of keratinization in the wound area and found that PBM can improve keratinization in secondary intention wound healing [17, 19, 20, 42]. In contrast, a study by Amorim et al. stated that there were no differences between the laser and control groups on any of the follow-ups for the amount of keratinized gingiva after gingivectomy. However, better-attached gingiva and clinical wound healing were observed, although it was not statistically significant [30]. Also, Lingamaneni et al. demonstrated that improved surface keratinization on the PBM site could not be achieved before 14th post-operative day [42].

In a study by Ozcelik, no statistically significant difference was observed between the degree of epithelialization areas in the laser and the control sites immediately after surgery. However, the intervention areas had greater epithelialization areas in comparison with the control sites at the following postoperative days [41]. Also, Vieira et al. found minor statistical significance in the PBM group for wound epithelialization after free gingival graft surgeries [40].

Ustaoglu et al. showed that tissue consistency and TT did not differ between PBM groups and controls at any time points. In contrast, the PBM group had better color matching as assessed by visual analog scale (VAS) scores compared to the control group [17].

Damante et al. (A) evaluated histologic features in wound areas that received PBM in one study and found no morphological or morphometric differences between laser and control groups. In another study by Damante et al. (B), photographs were taken for clinical evaluation. They reported that there was no advantage in using PBM to improve the wound healing outcome compared to the control group. A 670 nm diode laser was used in both studies [32, 33].

#### Post-operative pain and discomfort

The evaluated parameters regarding pain in the selected studies were mainly VAS scores and patient's pain response (NRS). Three studies showed pain relief after PBM [20, 34, 40], and two studies showed that PBM could not lead to pain relief in the wound area [19, 44].

Kohale et al. found that PBM can relieve pain at all evaluated time points (3, 7, 30 days) [20]. Also, Vieira et al. found that the VAS score for pain was lower in the PBM group from the first day to seventh, after free gingival graft surgery [40]. Isler et al. stated that although the control group had higher VAS scores at all time points, no significant differences were seen between the laser and control groups. The amount of systemic analgesic consumption did not vary between two groups. Also, patient discomfort was higher in the control group than the laser group on post-operative days [34]. In contrast, Heidary et al. found that during the first three hours post-surgery, the mean rate of VAS in the donor site was greater in the laser group in comparison with the control group. However, at longer evaluation time points, the groups did not show a substantial difference. Also, there was no difference in post-operative NSAIDs consumption between the groups [19]. Chawla et al. found that PBM cannot relieve post-operative pain in depigmentation procedures [44]. Another study by Ustaoglu et al. showed that the post-operative discomfort and the amount of analgesics did not vary through the 1st week post-surgery [17].

#### Meta-analysis

**Fig. 3:**
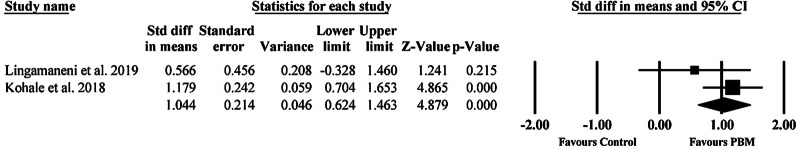
Forest plots of Landry wound healing index in the 7th postoperative day

Two studies were eligible to participate in the meta-analysis of the Landry wound healing index [20, 42]. The results of the analysis showed a statistically significant difference (p < 0.01) between PBM and control groups (SMD = 1.044 [95% CI 0.62–1.46]; p < 0.01) in the Landry wound healing index in the 7th post-operative day. The meta-analysis showed a large effect size and low heterogeneity (*I*^2^ = 28.9%) in favor of the positive effect of PBM on post-operative wound healing 7 days after surgery (Fig. 3), so the fixed effects model was used. As a publication bias test, Egger’s test was not appropriate in our meta-analysis because of the insufficient number of studies included (< 10) [46].

**Fig. 4:**
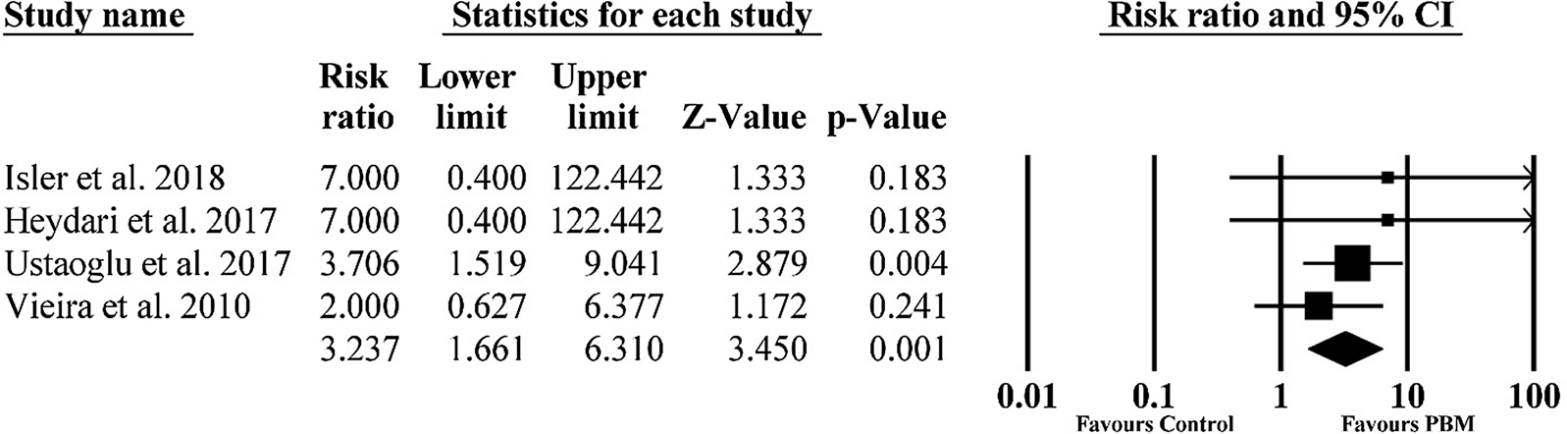
Forest plots of complete wound epithelialization in the 14th postoperative day

Also, four studies were eligible to participate in the meta-analysis of complete wound epithelialization [17, 19, 34, 40]. The results exhibited a statistically significant (p < 0.01) enhancement of epithelialization in the PBM group in comparison to the control group (RR = 3.23 [95% CI 1.66–6.31]; p < 0.01) on the 14th post-operative day. The result of the meta-analysis showed that when PBM was used, the odds of complete epithelialization was 3.2 times greater than without it. Also, there was almost no heterogeneity in the studies (*I*^2^ < 0.001%) favoring these results (Fig. 4). Random effects model was used in both analyses. As a publication bias test, Egger’s test was not appropriate in our meta-analysis because of the insufficient number of studies included (< 10) [46].

## Discussion

According to our search in the databases, this is the first systematic review and meta-analysis conducted to evaluate the effect of adjunctive use of PBM in periodontal surgical procedures leaving a secondary intention healing wound, such as gingivectomy, harvesting grafts from donor sites or depigmentation procedures, to assess its effectiveness on gingival healing and pain relief. All of the included studies were clinical trials, and the included non-randomized trials satisfied the pre-quality assessment.

Despite the clinical success of photobiomodulation, there are various, even contradictory theories about the actual mechanisms leading to improved clinical outcomes. The most popular and classic idea which has been challenged recently [21], is the absorption of red-to-near-infrared (R-NIR) photons by cytochrome c oxidase (COX) chromophores in cellule’s mitochondria that does a pivotal part in the photon-cellule interaction. The absorption process stimulates the electrons in chromophores, creating a proton gradient and ultimately leading to an increase in ATP production and glycolysis leading to higher cellular proliferation and differentiation [22]. Several studies have indicated that PBM can facilitate the speed and quality of wound healing and different mechanisms have been investigated. Keskiner et al. reported an increase in palatal wound fluid (PWF), transforming growth factor-b1 (TGF-b1), platelet-derived growth factor-BB (PDGF-BB), and interleukin-8 (IL-8) levels. This might indicate an increased rate of wound healing by stimulation of the secretion of selected mediators [43]. Enhanced collagen production, increased levels of growth factors and extracellular matrix-remodeling proteins, stimulated synthesis of adenosine triphosphate, fibroblastic proliferation, and angiogenesis, in a dose-dependent manner have also been reported [16, 17, 19, 20]. It can be assumed that improved pain relief, re-epithelialization, and tissue thickness could be the direct impact of the improved wound healing process.

In a recent meta-analysis, it was stated that the mechanism and effect of PBM on primary or secondary wound healing might be significantly different [47]. This might be due to different healing mechanisms and cellular and molecular events between secondary and primary wound healing. Secondary healing involves more granulation and collagenous tissue formation in the proliferation stage, and a higher amount of remodeling and contraction in the remodeling stage of wound healing. Moreover, secondary healing is associated with more tendency to wound infection and leaves more scar tissue in the wound site [8]. Therefore, the application of PBM may more beneficial in these patients. In this study, we have only assessed the effect of PBM on secondary intention healing gingival wounds.

Although, all of the included studies in the present review have used irradiation wavelengths in the red and near infra-red range; they showed a great variation in irradiation parameters and the method of application of the adjunctive PBM therapy, making it challenging to draw evidence-based conclusions regarding the most appropriate irradiation settings needed for improvement in healing and pain relief. The most suitable laser settings for biostimulation of healing and reduction of post-operative pain of periodontal surgical wounds have not been determined yet due to the great variation observed in irradiation parameters in the available literature. Further studies with similar designs are needed to add evidence for evidence based conclusions. Factors, such as the diameter of the fiber, can alter power density and energy output in the application of lasers. It could also change the quantity of energy that is applied during the treatment, altering the wound-healing effect of PBM.

The included studies utilized various wavelengths and irradiation parameters for PBM of the wounds. Only one study used an LED 650 nm device reporting favourable effects on both wound healing and post-operative pain, and in one study PBM was performed using an Nd:YAG laser (1084 nm) device which had positive effects on healing. The other included studies used diode lasers with red to near infra-red wavelengths (588–970 nm) for irradiation of the surgical sites. Energy densities ranged from 1.6 to 8.6 J/cm^2^. The majority of studies applied an energy density of 4 J/cm^2^ per point. However, the output powers ranged from as low as 0.05–5 W.

In the present study, despite all methodological variations, the results of the meta-analysis of the Landry wound healing index and complete wound epithelialization demonstrated a statistically significant improvement in secondary wound healing after periodontal surgeries. One of the studies from which the meta-analysis of the Landry wound healing index was conducted, had a very high quality with no risk of bias [42]. The other study did not blind the outcome assessor and had detection bias with lower quality [20]. Four studies were used for the meta-analysis of the complete wound epithelialization and only one of them had high quality with low risk of bias [34]. One had incomplete outcome data (attrition bias) [19], one study had not blinded the participants or personnel (performance bias) and also had some selective reporting (reporting bias) [17], and one had issues with randomization process (selection bias) and blinding of the participants or personnel (performance bias) with relatively low quality [40].

Based on the included studies, it appears that PBM can be beneficial in improving secondary wound healing after certain types of periodontal surgeries. However, the included studies showed some controversies about the efficiency of PBM on post-operative pain. These results may be due to two factors: Firstly, pain measurement is subjective. Secondly, although the VAS scale is a valid method, the range of results is widely heterogeneous [48]. Moreover, the method of pain sensation evaluation varied in the studies. For example, some of the studies used external stimuli to measure pain. However, a recent systematic review on photobiomodulation and acute pain has indicated positive results for PBM and reported similar effects to NSAIDs consumption [49].

The summary of the risk of bias evaluation is shown in Fig. 2. The main source of bias in the included studies was the performance bias, which shows that most of the studies did not focus on blinding the participants and personnel. To reduce this type of bias, the researchers could use sham lasers in the control sites. Or they could use the same laser in the control sites without pressing the button just to mimic the application of PBM. Moreover, to blind the operator; a person not involved in the study design could be asked to activate the laser in the specified sites.

Another main source of bias was the selection bias. Random sequence and concealment of allocation are considered of great importance in any study. Future studies should pay more attention to these risks in their studies.

### Study limitations

In the present study, we did not include studies in which the patients had specific risk factors such as smoking or diabetes as there was not enough data on possible systemic complications and PBM therapy. Also, due to incomplete information and methodological heterogeneity, variable laser parameters, and methods of its application, the authors couldn't perform a meta-analysis for all of the variables in the included studies. Regarding post-operative pain and discomfort, considerable heterogeneity existed among the evaluation methods. For instance, in some studies, VAS was evaluated by application of an external stimuli like air spray [48, 50], while in other studies no stimulation method was utilized. One of the included studies used the NRS index to evaluate post-operative pain [20]. Overall, because of the different methods used to evaluate post-operative pain in these studies, the criteria for a meta-analysis were not met.

Furthermore, when assessing the tissue epithelialization, the existing diversity in the used methods like the evaluation of pictures taken from intervention areas [30], visual inspection of the wound [17, 20, 34, 40, 41], or the use of computer software [44] did not allow us to perform a meta-analysis. The exact area of the initial wounds was not mentioned in any of the studies, which may be an interesting factor to consider in future study designs evaluating the effect of PBM in wound healing.

## Conclusion

Based on the results of the current systematic review, it may be suggested that the application of PBM is a beneficial adjunct to promote second intention wound healing in periodontal soft tissue surgeries.

Currently, no optimal laser application settings can be suggested due to the extensive heterogeneity of laser parameters and variable study designs. Studies with a low risk of bias, especially in randomization and blinding, are needed to produce high-quality evidence. Also, further studies using comparable irradiation criteria with larger sample sizes and longer follow-ups on a similar procedure are necessary to indicate which parameters have essential roles in using PBM to accelerate the secondary intention healing in gingival wounds.

## Supplementary Information

**Additional file 1.** Search strategies of the study.

**Additional file 2.** Excluded full-text titles with related reasons.

## Data Availability

All data supporting the conclusions of this article are included within the article (and its additional files).

## Abbreviations

PBM: Photobiomodulation
LLLT: Low-level laser therapy
LED: Light emitting diode
CAL: Clinical attachment level
PD: Probing depth
WHI: Wound healing Index
RR: Relative risk
MD: Mean differences
HI: Healing index
CH: Clinical healing
TT: Tissue thickness
RWA: Remaining wound area
VAS: Visual analog scale
NSAIDs: Nonsteroidal anti-inflammatory drugs
PWF: Palatal wound fluid
TGF-b1: Transforming growth factor-b1
PDGF-BB: Platelet-derived growth factor-BB
IL-8: Interleukin-8
R-NIR: Red-to-near-infrared
COX: Cytochrome c Oxidase
ATP: Adenosine triphosphate
