# Supplementary material for: Effect of photobiomodulation in secondary intention gingival wound healing—a systematic review and meta-analysis
Source: BMC Oral Health. 2021 May 13;21:258. doi: 10.1186/s12903-021-01611-2 (PMC8120828; doi:10.1186/s12903-021-01611-2)
Supplement: Supplementary file 1 — Additional file 1. Search strategies of the study. [file 12903_2021_1611_MOESM1_ESM.docx]

**Appendix 1. Search Strategies of the Study.**

**Pubmed**

(((((((((("Low-Level Light Therapy"[Mesh]) OR (photobiomodulation[Text Word])) OR (lllt[Text Word])) OR ('low power laser'[Text Word])) OR ('low intensity laser'[Text Word])) OR ('low power laser'[Text Word])) OR ('laser biostimulation'[Text Word])) OR ('low energy laser'[Text Word])) OR ('low level laser'[Text Word])) OR ('low level light'[Text Word])) AND (((((((periodontal surger*[Text Word]) OR ("Gingivectomy"[Mesh])) OR (Gingivectomy[Text Word])) OR (pigmentation[Text Word])) OR ("Hyperpigmentation"[Mesh])) OR (depigmentation[Text Word])) OR (palat*[Text Word] AND donor[Text Word]))

**Embase**

('low level laser therapy'/exp OR 'photobiomodulation'/exp OR 'photobiomodulation':ti,ab OR 'lllt':ti,ab OR 'low level laser therapy':ti,ab OR 'low intensity laser':ti,ab OR 'low power laser':ti,ab OR 'laser biostimulation':ti,ab OR 'low energy laser':ti,ab OR 'low level laser':ti,ab OR 'low level light':ti,ab) AND ('gingivectomy'/exp OR 'Gingivectomy':ti,ab OR 'pigmentation':ti,ab OR 'Hyperpigmentation':ti,ab OR 'depigment*':ti,ab OR ('palat':ti,ab AND 'donor':ti,ab))

**Scopus**

( TITLE-ABS-KEY ( "Low Level Light Therapy"  OR  "photobiomodulation"  OR  "lllt"  OR  "'low power laser'"  OR  "low intensity laser"  OR  "low power laser"  OR  "laser biostimulation"  OR  "low energy laser"  OR  "low level laser"  OR  "low level light" ) )  AND  ( TITLE-ABS-KEY ( "periodontal surger*"  OR  "Gingivectom*"  OR  "pigmentation"  OR  "gingiva* Hyperpigmentation"  OR  "depigment*"  OR  ( palat*  AND  donor ) ) )

**Web of Science**

---------------------------------------------------------------#1--------------------------------------------------------------------------
TS=('Low Level Light Therapy' OR 'photobiomodulation' OR 'lllt' OR 'low power laser' OR 'low intensity laser' OR 'low power laser' OR 'laser biostimulation' OR 'low energy laser' OR 'low level laser' OR 'low level light')

---------------------------------------------------------------#2--------------------------------------------------------------------------

TS=('periodontal surger*' OR Gingivectom* OR pigmentation OR depigment*OR (palat* AND donor) OR 'gingiva* Hyperpigmentation')

 ---------------------------------------------------------------#3--------------------------------------------------------------------------

#2 AND #1

**PROQUEST**

ab("Low-Level Light Therapy" OR "photobiomodulation" OR "lllt" OR "low power laser" OR "low intensity laser" OR "low power laser" OR "laser biostimulation" OR "low energy laser" OR "low level laser" OR "low level light") AND ab("periodontal surger*" OR "Gingivectom*" OR "pigmentation" OR "gingiva* Hyperpigmentation" OR "depigment*" OR ( palat* AND donor ))
