# Supplementary material for: Effect of photobiomodulation in secondary intention gingival wound healing—a systematic review and meta-analysis
Source: BMC Oral Health. 2021 May 13;21:258. doi: 10.1186/s12903-021-01611-2 (PMC8120828; doi:10.1186/s12903-021-01611-2)
Supplement: Supplementary file 2 — Additional file 2. Excluded full-text titles with related reasons. [file 12903_2021_1611_MOESM2_ESM.docx]

| **Study Title** | **AUTHOR (YEAR)** | **REASON FOR EXCLUSION** |
| --- | --- | --- |
| Early assessment of wound healing, pain and patient's discomfort after free gingival graft surgery for widening the zone of attached gingiva and the shallow vestibule of mouth with the use of various healing biostimulative methods | Wiench R. et al. (2016) | no secondary intention wound healing evaluation (examining the recipient site) |
| Effect of GaAIAs low-level laser therapy on the healing of human palate mucosa after connective tissue graft harvesting: randomized clinical trial | Dias S. B. et al. (2015) | no secondary intention wound healing evaluation (palatal donor sites were sutured) |
| Comparison of two power densities on the healing of palatal wounds after connective tissue graft removal: randomized clinical trial | da Silva Neves F. L. et al. (2016) | no secondary intention wound healing evaluation (palatal donor sites were sutured) |
| The effects of low level laser irradiation on gingival inflammation | Pejcic A. et al. (2010) | no secondary intention wound healing evaluation (periodontal therapy, not assessing a wound) |
| Effect of Laser Bio-Stimulation on Postoperative Pain and Tissue Response | Khanna D. B. D. S. M. D. S. et al. (2017) | no secondary intention wound healing evaluation (flap surgery) |
| Comparison of laser periodontal treatments with conventional periodontal treatments in patients with advanced periodontitis | Gaspirc B. et al. (2012) | Not PBM |
| Efficacy of Low-level Laser Therapy, Hyaluronic Acid Gel, and Herbal Gel as Adjunctive Tools in Gingivectomy Wound Healing: A Randomized Comparative Clinical and Histological Study | Reddy S.P. et al. (2019) | No control group (quasi-experimental study) |
| Evaluation of the effect of 660 nm low power laser on pain and healing in palatal donor site: a randomized controlled clinical trial | Moslemi N. et al. (2014) | not in English |
